# Inflammatory microenvironment of fibrotic liver promotes hepatocellular carcinoma growth, metastasis and sorafenib resistance through STAT3 activation

**DOI:** 10.1111/jcmm.16256

**Published:** 2021-01-07

**Authors:** Yuchuan Jiang, Peng Chen, Kaishun Hu, Guanqi Dai, Jinying Li, Dandan Zheng, Hui Yuan, Lu He, Penghui Xie, Mengxian Tu, Shuang Peng, Chen Qu, Wenyu Lin, Raymond T. Chung, Jian Hong

**Affiliations:** ^1^ Department of Abdominal Surgery Integrated Hospital of Traditional Chinese Medicine Southern Medical University Guangzhou China; ^2^ Guangdong Provincial Key Laboratory of Malignant Tumor Epigenetics and Gene Regulation Medical Research Center Sun Yat‐Sen Memorial Hospital Sun Yat‐Sen University Guangzhou China; ^3^ Department of Gastroenterology Guangzhou Overseas Chinese Hospital Jinan University Guangzhou China; ^4^ Department of Radiotherapy Affiliated Cancer Hospital & Institute of Guangzhou Medical University Guangzhou China; ^5^ Department of Pathophysiology School of Medicine Jinan University Guangzhou China; ^6^ Liver Center and Gastrointestinal Division Massachusetts General Hospital Harvard Medical School Boston MA USA

**Keywords:** hepatic inflammatory microenvironment, hepatocellular carcinoma, intrahepatic metastasis, sorafenib resistance

## Abstract

The pro‐inflammatory and pro‐fibrotic liver microenvironment facilitates hepatocarcinogenesis. However, the effects and mechanisms by which the hepatic fibroinflammatory microenvironment modulates intrahepatic hepatocellular carcinoma (HCC) progression and its response to systematic therapy remain largely unexplored. We established a syngeneic orthotopic HCC mouse model with a series of persistent liver injury induced by CCl_4_ gavage, which mimic the dynamic effect of hepatic pathology microenvironment on intrahepatic HCC growth and metastasis. Non‐invasive bioluminescence imaging was applied to follow tumour progression over time. The effect of the liver microenvironment modulated by hepatic injury on sorafenib resistance was investigated in vivo and in vitro. We found that the persistent liver injury facilitated HCC growth and metastasis, which was positively correlated with the degree of liver inflammation rather than the extent of liver fibrosis. The inflammatory cytokines in liver tissue were clearly increased after liver injury. The two indicated cytokines, tumour necrosis factor‐α (TNF‐α) and interleukin‐6 (IL‐6), both promoted intrahepatic HCC progression via STAT3 activation. In addition, the hepatic inflammatory microenvironment contributed to sorafenib resistance through the anti‐apoptotic protein mediated by STAT3, and STAT3 inhibitor S3I‐201 significantly improved sorafenib efficacy impaired by liver inflammation. Clinically, the increased inflammation of liver tissues was accompanied with the up‐regulated STAT3 activation in HCC. Above all, we concluded that the hepatic inflammatory microenvironment promotes intrahepatic HCC growth, metastasis and sorafenib resistance through activation of STAT3.

## 1 INTRODUCTION

Hepatocellular carcinoma (HCC) is ranked as the sixth most common neoplasm and the third leading cause of cancer death.^1^ More than 50% of patients are diagnosed with advanced disease.^2^, ^3^ HCC is aggressively malignant and highly invasive, which can lead to the death of patients mainly due to rapid progression of intrahepatic tumour.^4^, ^5^ In addition, systemic therapies for advanced HCC have limited efficacy with a median survival of approximately 12 months.^6^, ^7^ Although some new systemic drugs (lenvatinib, regorafenib, cabozantinib and ramucirumab) have recently been approved by the Food and Drug Administration (FDA) for HCC treatment, sorafenib remains the standard of care for frontline therapy.^6^ However, sorafenib‐acquired resistance has resulted in unfavourable survival benefits.^8^ Therefore, clarify study of the underlying mechanisms associated with intrahepatic HCC progression and sorafenib resistance is urgently need.

Most cases of HCC arise in fibrotic or cirrhotic livers which is resulted from the chronic liver disease, hepatitis B or C virus infections, alcoholic liver disease and non‐alcoholic fatty liver disease,^9^, ^10^ and is characterized by persistent hepatic injury and chronic unresolved inflammation.^11^, ^12^ Multiple inflammatory cytokines during chronic liver disease, such as interleukin‐6 (IL‐6) and tumour necrosis factor‐α (TNF‐α), produced by injured hepatocytes and macrophages contribute to neoplastic transformation of hepatocytes (hepatocarcinogenesis).^2^, ^12^ Nevertheless, organ inflammation impacts all stages of malignancy from tumorigenesis to growth and metastatic progression, as well as the effectiveness of therapy.^13^ Other studies found that tumour growth could be inhibited by reducing pro‐inflammatory cytokines and decreasing the infiltration of neutrophils of colon tissue in mouse model with colitis‐associated colorectal cancer,^14^ and chronic pancreatitis contributes to pancreatic ductal adenocarcinoma metastasis and resistance to therapies via stimulating the epithelial‐mesenchymal transition (EMT) and amplifying Ras activity.^15^ However, the studies on the effect of hepatic inflammatory microenvironment in intrahepatic HCC progression and drug resistance are still limited.^10^, ^11^

Signal transducer and activator of transcription 3 (STAT3) is a transcription factor that regulates the injury‐inflammation‐regeneration response. In normal tissues, STAT3 activation is highly regulated and transient by phosphorylation.^16^ However, constitutively activated STAT3 was detected in approximately 60% of human HCC specimens and phosphorylated STAT3 (p‐STAT3) positive tumours were more aggressive^11^ In addition, activated STAT3 of HCC impairs sorafenib‐induced cell death by promoting anti‐apoptotic protein or cancer stem cell markers.^17^, ^18^ Although it has been reported that STAT3 inhibitor enhanced the sensitivity of HCC cells with high expression of p‐STAT3 to sorafenib in vitro, and STAT3 activation in malignant cells depends on signals produced by neighbouring cells,^13^ the effect and mechanism of constitutive STAT3 activation has not been fully understood in vivo.^11^, ^19^

Here, we established a syngeneic orthotopic HCC mouse model which recapitulates the inflammatory microenvironment of fibrotic liver. Our results indicated that liver inflammation exerts favourable effects on HCC progression and sorafenib resistance by STA3 activation. Consistently, the STAT3 inhibitor S3I‐201 attenuated HCC growth and metastasis within the inflammatory microenvironment and significantly improved the sorafenib efficacy. It was also found that the remarkable liver inflammation of human liver is accompanied with p‐STAT3 up‐regulation in HCC.

## 2 MATERIALS AND METHODS

### 2.1 Cell culture and infectious virus

Mouse HCC cell line Hepa1‐6, Human HCC cell lines Huh7 and Hep3B were cultured in high‐glucose Dulbecco's Modified Eagle Medium (Gibco, NY, USA) supplemented with 10% foetal bovine serum (Gibco), 100 U/mL penicillin and 100 U/mL streptomycin, and incubated at 37°C under an atmosphere containing 5% CO_2_. The RFP‐labelled luciferase reporter‐expressing Hepa1‐6 cells were obtained by transduction with the retroviral vector.

### 2.2 Reagents and chemicals

Recombinant human TNF‐α and IL‐6 were purchased from PeproTech. S3I‐201 (NSC 74859) and sorafenib (BAY 43‐9006) were purchased from Selleck Chemicals. For in vitro experiments, S3I 201 and sorafenib were dissolved in DMSO (Sigma‐Aldrich) and further diluted to the required concentration. For in vivo experiments, S3I‐201 and sorafenib suspension were prepared in 0.5% carboxymethyl cellulose sodium normal saline solution. Antibodies to STAT3, phospho‐STAT3 (p‐STAT3), E‐cadherin, Vimentin, N‐cadherin, Mcl‐1, Cleaved PARP and glyceraldehyde 3‐phosphate dehydrogenase (GAPDH) were purchased from Cell Signaling Technology. Fluorescein isothiocyanate (Alexa Fluor^®^ 555)‐labelled goat anti‐rabbit IgG secondary antibody was purchased from invitrogen.

### 2.3 Establishment of an orthotopic mouse model of HCC with chronic liver injury

4‐ to 6‐week‐old male C57BL/6 mice were induced with varying degrees of chronic liver injury by CCl_4_ (40% in 100 μL olive oil/ mouse, v/v) gavage for 4, 6 and 10 weeks,^20^, ^21^ and age‐matched mice administered with olive oil (100 μL/mouse) served as the control group. Following that, mice were injected by 25 μL of HCC cell/Matrigel solution (containing 1 × 10^6^ Hepa1‐6 cells) in the subcapsular region of the liver and then killed at 10 days post‐tumour cell implantation or humane end point. The tumour progression was monitored by In an Vivo Imaging System (FX pro, Bruker) indicated by bioluminescence intensity. Tumour and matched non‐tumour liver tissues were collected for further examination and analysis. The mice were maintained in the laboratory for animal experimentation in a specific pathogen‐free environment with laminar air‐flow conditions, a 12‐hour light‐dark cycle and at a temperature of 22°C‐25°C. All animals had free access to standard laboratory mouse food and water. Animal experiments were approved by the Bioethics Committee of Southern Medical University and were performed according to the established guidelines.

### 2.4 In vivo treatment studies

C57BL/6 mice were subjected to CCl_4_ gavage for 6 weeks in the chronic liver injury group and administered with olive oil in the normal liver group. Then, all mice were implanted with Hepa1‐6 cells. For SI3‐201 monotherapy, mice were divided into three groups (n = 8): normal liver group, liver injury group and treatment group. For sorafenib monotherapy, mice were divided into four groups: vehicle (normal liver), sorafenib treatment (normal liver), vehicle (chronic liver injury) and sorafenib treatment (chronic liver injury). For combination treatment of sorafenib and SI3‐201, C57BL/6 mice were treated by CCl_4_ gavage for 6 weeks and implanted with hepa1‐6 cells. These mice were randomized into four groups (n = 8): vehicle, SI3‐201, sorafenib, and combination therapy of sorafenib and SI3‐201. On day 3 following tumour cell inoculation, sorafenib was administered orally at the dose of 30 mg/kg and SI3‐201 was injected intraperitoneally at the dose of 5 mg/kg for 7 days. Mice were killed 10 days after HCC implantation.

### 2.5 Patients and specimens

Forty‐seven liver samples from HCC patients who underwent hepatectomy were collected in China. All liver samples were obtained under protocols approved by the Integrated Hospital of Traditional Chinese Medicine of Southern Medical University Office for Protection of Human Subjects.

### 2.6 Statistical analysis

The Student *t* test was used to compare values between subgroups. Overall survival (OS) was calculated by Kaplan‐Meier survival analysis and log‐rank tests. The Pearson correlation test (two‐tailed) was used to calculate the correlation coefficient. Data were expressed as mean ± standard deviation (SD) of at least three biological replicates. Statistical significance was declared if *P* < .05. All analyses were performed using SPSS software (Version 23.0, IBM).

For further details regarding the methods and materials, please refer to Appendix S1.

## 3 RESULTS

### 3.1 Severe chronic liver injury led to rapid HCC progression in a mouse model

**FIGURE 1:**
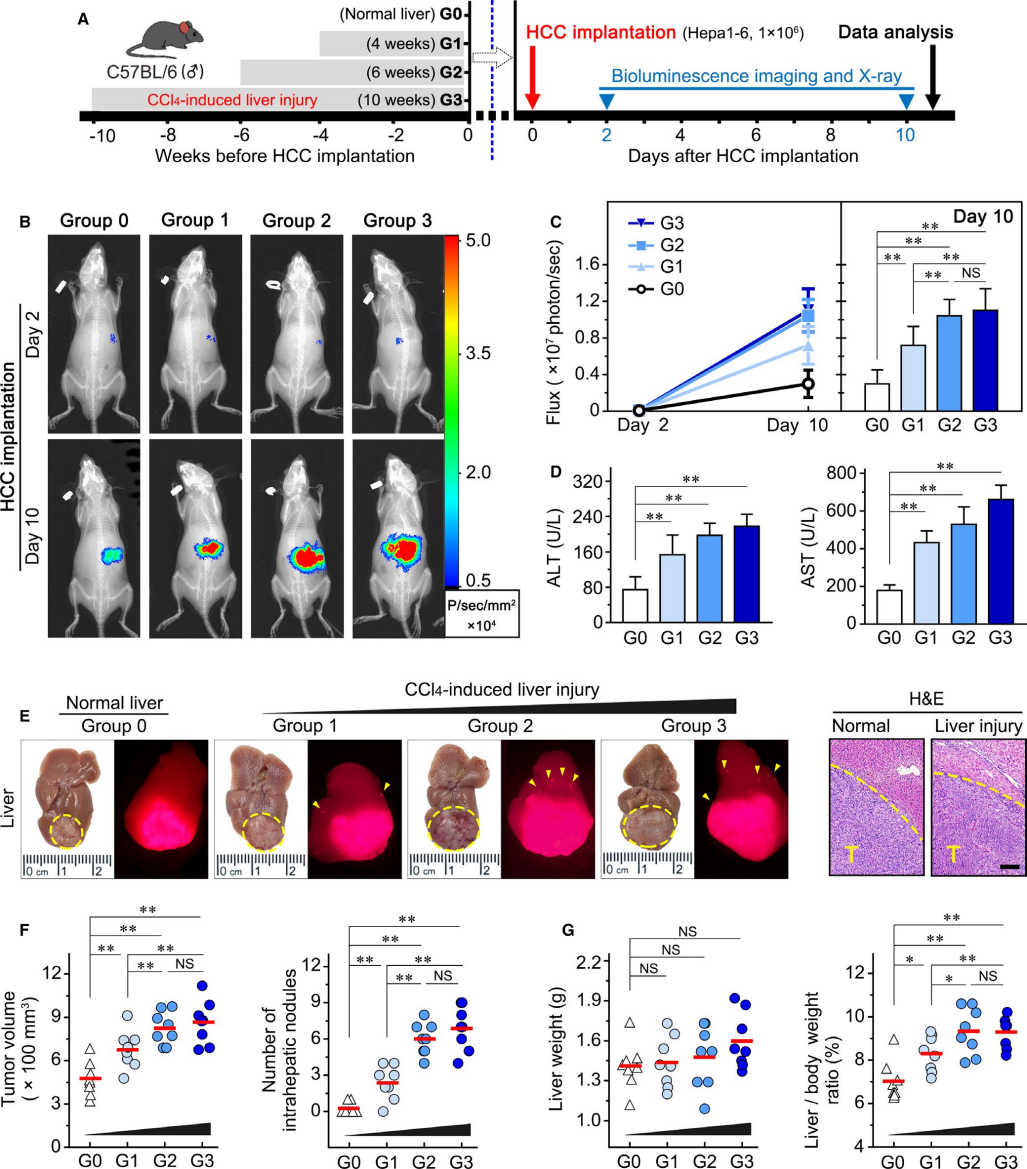
Increased chronic liver injury resulted in a rapid HCC progression in a mouse model. A, Experimental design for comparison of HCC progression in normal liver (Group 0, G0) and varying degrees of injured liver (Group 1, G1; Group 2, G2; and Group 3, G3). Mice bearing tumours detectable by bioluminescence were killed at 10 d after HCC implantation (n = 8/group). (B) The representative bioluminescence images of four groups (G0‐G3) were shown at the two indicated times. (C) Total bioluminescence flux were measured and shown as the mean with standard deviation (SD). (D) Serum levels of ALT and AST in four groups. (E) The tumours were compared by both bright field and fluorescence. The circle indicated primary tumour and the arrow indicated intrahepatic metastases (Left panel). The tumorigenesis were confirmed by H&E staining (right panel). (F) Tumour volume per liver (100 mm^3^) and number of intrahepatic nodules were calculated. (G) Tumour burden was further evaluated by liver weight and liver to body weight ratio. Scale bars, 200 μm. Data presented are means ± SD. NS, not significant. *P < .05; **P < .01

To investigate the effect of hepatic pathologic microenvironment on HCC growth and metastasis, we established a syngeneic orthotopic HCC mouse model with a series of chronic liver injury (Figure 1A). We observed the increased bioluminescence signal on day 10 compared to day 2 after tumour implantation, indicating HCC progression in vivo (Figure 1B). Further analysis demonstrated that the bioluminescence intensity of liver injury groups (G1, G2 and G3) was stronger than that of the normal liver group (G0) on day 10 (all *P* < .01). In addition, longer CCl_4_ administration of 6 or 10 weeks compared to 4 weeks (G2 vs G1: *P* = .005; G3 vs G1: *P* = .004) resulted in a stronger bioluminescence intensity in vivo (Figure 1C). However, there was no significant difference in bioluminescence intensity between the liver injury groups for 6 and 10 weeks (G2 vs G3: *P* = .579; Figure 1C). The elevating level of serum alanine aminotransferase (ALT) and aspartate aminotransferase (AST) among groups 1‐3 indicated the severity of chronic liver injury by CCl_4_ gavage (Figure 1D).

Further, we measured the tumour volume and intrahepatic metastasis by fluorescent imaging (Figure 1E). Consistently, the mouse hepatoma in liver injury groups (G1, G2 and G3) exhibited accelerated growth and metastasis compared to the normal liver group (G0) (all *P* < .01). Moreover, the mice with liver injury for 6 and 10 weeks compared to 4 weeks have the larger tumours (G2 vs G1: *P* = .029; G3 vs G1: *P* = .018) and greater numbers of intrahepatic metastasis (G2/G3 vs G1: both *P* < .01; Figure 1F). The tumour volume and metastatic nodules were not significantly different between the liver injury groups for 6 and 10 weeks (Figure 1F). Liver/body weight ratio showed the same tendency (Figure 1G).

Consistent with the previous studies,^22^, ^23^ our results indicated that chronic liver injury contributed to HCC growth and intrahepatic metastasis. Importantly, we found that the intrahepatic tumour progression was further accelerated by greater severity of chronic liver injury in an orthotopic HCC mouse model, suggesting the dynamic effect of hepatic pathology microenvironment on HCC promotion.

### 3.2 HCC progression is closely associated with the degree of liver inflammation

**FIGURE 2:**
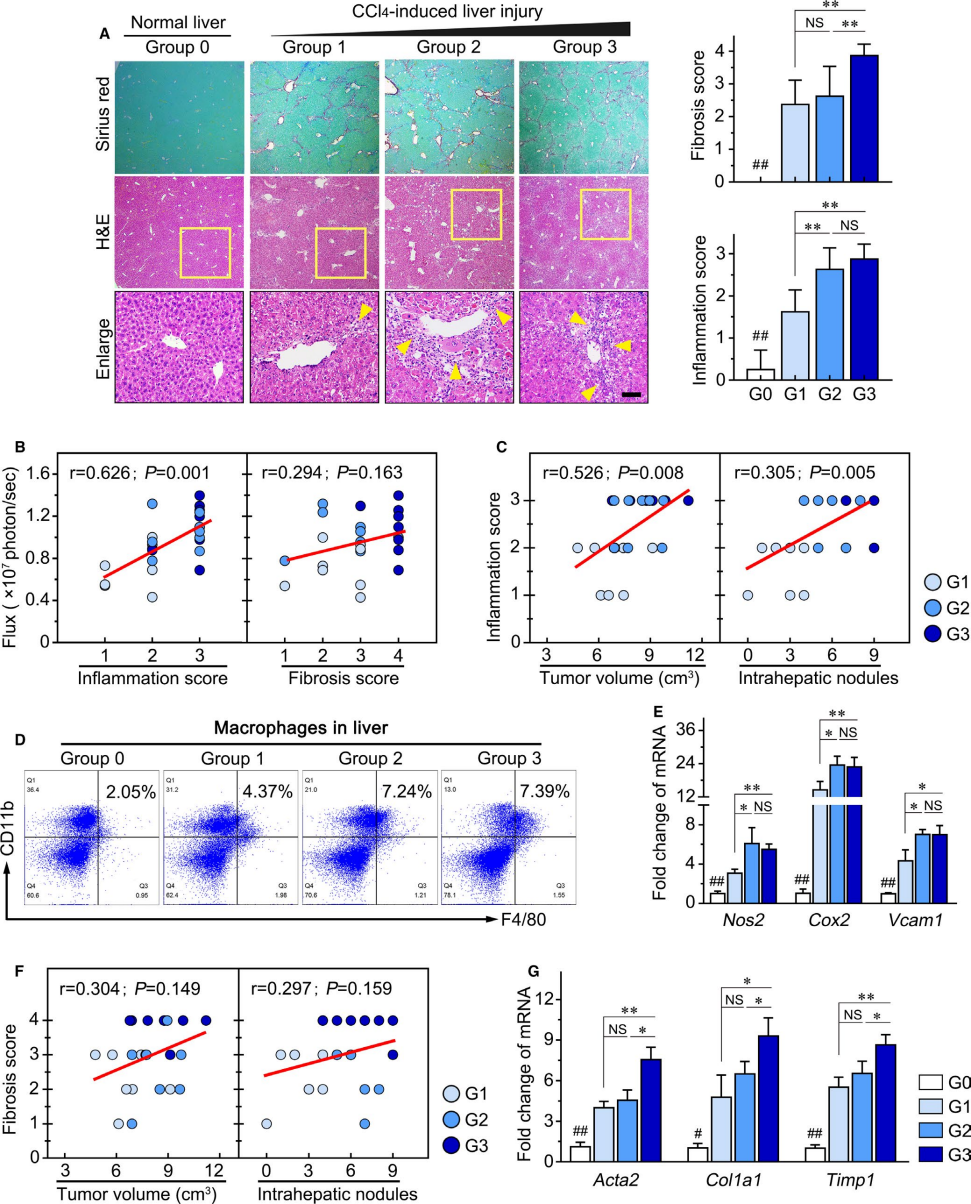
The HCC progression is significantly related to the degree of liver inflammation. A, Representative images of continuous H&E and Sirius red staining of liver tissue in the normal liver group (G0) and injured liver groups (G1, G2 and G3). The arrow indicates the periportal infiltration of inflammatory cells (Left panel). Liver fibrosis and inflammation score in four groups (Right panel). In liver injury groups (G1, G2 and G3), the degree of liver inflammation was positively correlated (B) bioluminescence intensity, (C) tumour volume and the number of intrahepatic metastasis. D, Liver‐infiltrating macrophage and (E) inflammation‐related genes were detected by flow cytometry and q‐PCR. F, The extent of liver fibrosis had no significant correlation with the tumour growth and intrahepatic metastasis. G, The extent of liver fibrosis was verified by detecting fibrosis‐related genes in the liver injury group. Scale bars, 100 μm. Data presented are means ± SD. NS, not significant. *P < .05; **P < .01; #*P *< .05 and ##*P *< .01 vs liver injury group

Chronic liver injury resulted in the hepatic pathologic microenvironment characterized by non‐resolving inflammation and fibrosis.^11^ Thus, we evaluated the relationships between liver inflammation or fibrosis and HCC progression. Distinct collagen bridges and periportal inflammation were presented in liver injury groups (G1, G2 and G3) compared to the normal liver group (G0) (all *P* < .01; Figure 2A). In addition, the fibrosis score manifested greater extent of liver fibrosis in the liver injury group for 10 weeks than in 4 and 6 weeks (G3 vs G1: *P* < .001; G3 vs G2: *P* = .002), while higher inflammation scores were found in liver injury groups for 6 and 10 weeks than 4 weeks (G2 vs G1: *P* < .001; G3 vs G1: *P* = .001; Figure 2A). We further analysed the correlation between bioluminescence intensity and liver inflammation or fibrosis score in the liver injury group, and found that bioluminescence intensity was more closely correlated with the degree of liver inflammation (*r* = 0.626, *P* = .001) than the extent of liver fibrosis (*r* = 0.294, *P* = .163; Figure 2B).

In addition, tumour volume (*r* = 0.526, *P* = .008) and the number of intrahepatic metastasis (*r* = 0.305, *P* = .005) also exhibited significantly positive correlation with the inflammation degree in liver injury groups (Figure 2C). We confirmed the degree of liver inflammation by examining hepatic macrophages and inflammation‐related genes (*Nos2*, *Cox2* and *Vcam1*).^24^ The results indicated that the proportion of hepatic macrophages (Figure 2D) and the expression of inflammation‐related genes (Figure 2E) were higher at 6 and 10 weeks compared to 4 weeks of liver injury (G2/G3 vs G1), which was consistent with results indicated by inflammation score. Moreover, analysis demonstrated that the extent of liver fibrosis had less correlation with the intrahepatic HCC progression (tumour volume and the intrahepatic metastasis) relative to the degree of liver inflammation in liver injury groups (Figure 2F). The level of fibrosis‐related genes (*Acta2*, *Col1a1* and *Timp1*) was increased in liver injury groups for 10 weeks compared to 4 and 6 weeks, which confirmed the extent of liver fibrosis evaluated by fibrosis score (Figure 2G). These data suggested that liver inflammation induced by chronic liver injury exerts favourable effects to intrahepatic HCC progression.

### 3.3 Pro‐inflammatory cytokines derived from liver microenvironment enhance HCC growth and metastasis by the activation of STAT3

**FIGURE 3:**
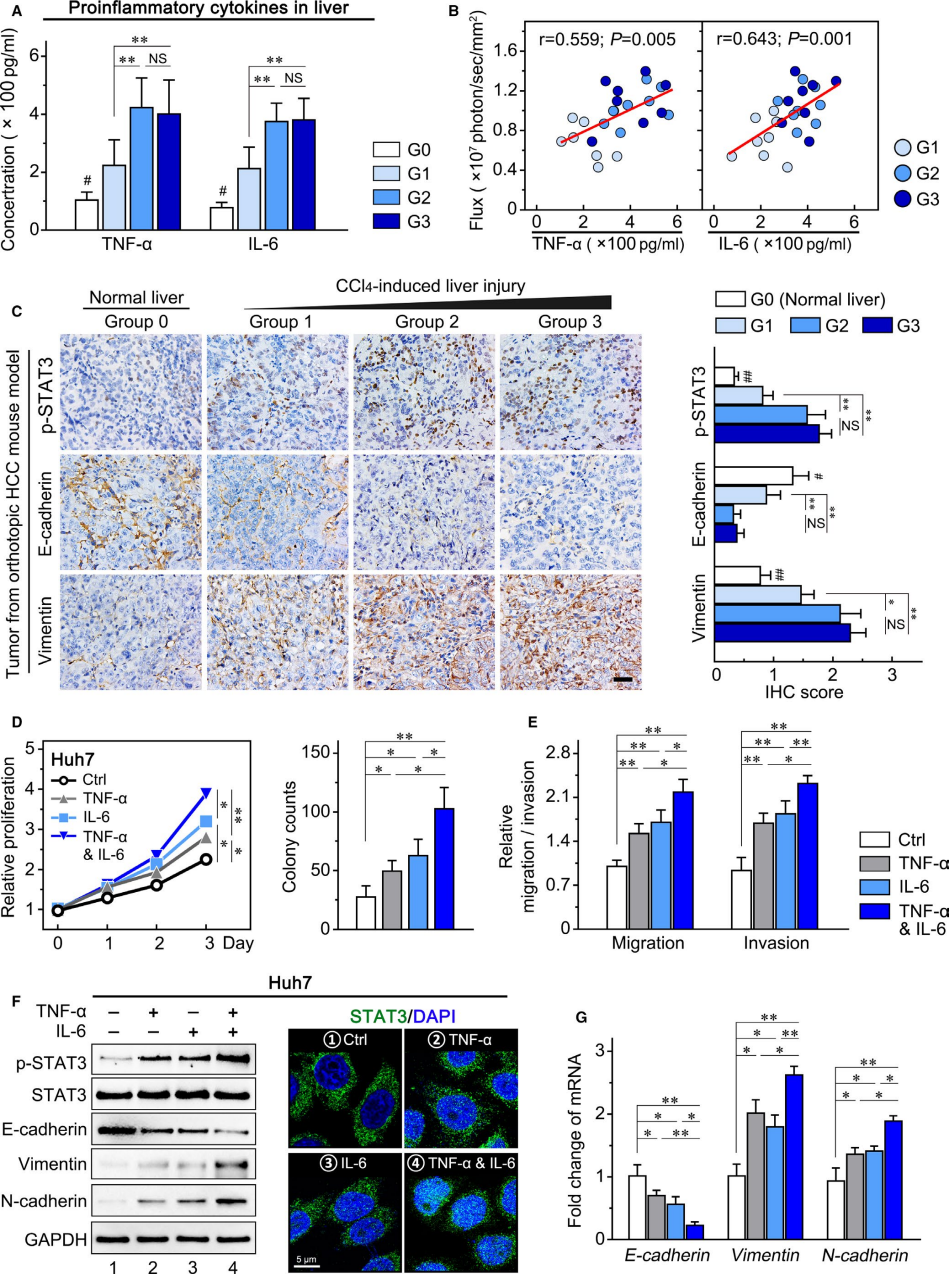
Inflammatory cytokines, TNF‐α and IL‐6, in liver tissues promote HCC proliferation and invasion by combined activation of the STAT3/EMT pathway. The level of TNF‐α and IL‐6 in liver tissues of four groups (G1, G2 and G3) examined by (A) ELISA was (B) positively associated with bioluminescence intensity. C, Tumour sections from orthotopic HCC mouse model were stained for p‐STAT3, E‐cadherin and Vimentin. After individual and combined treatment with exogenous TNF‐α (40 ng/mL) and IL‐6 (20 ng/mL), (D) Huh7 proliferation was measured by CCK8 assays and monolayer colony formation assays; (E) Transwell assays were performed to determine the migration and invasion ability of Huh7 cells; (F) Expression of STAT3, p‐STAT3 and EMT‐related markers were detected in Huh7 cells (left panel), the process of transnuclear trafficking of phosphorylated STAT3 in Huh7 cells were captured by confocal microscopy. The STAT3 signal was stained with anti‐total STAT3 antibody (green colour), and the nucleus was stained with DAPI (blue colour); (G) The mRNA‐level fold change of EMT‐related markers in Huh7 treated with indicated cytokines. Scale bars, 100 μm. Data presented are means ± SD. NS, not significant. *P < .05; **P < .01; #*P *< .05, vs liver injury group

As the inseparable component of liver inflammation, the pro‐inflammatory cytokines are the driving force of the inflammatory response.^25^ We firstly detected the level of major pro‐inflammatory cytokines in liver tissues by q‐PCR, including TNF‐α, IL‐1β, IL‐6, IL‐8, IL‐12, IL‐17 and IL‐18, and found that these cytokines were significantly up‐regulated in the liver injury group (data not shown). It has been reported that TNF‐α or IL‐6 promotes tumour proliferation and invasion in an autocrine‐dependent manner.^2^, ^26^ Thus, we further used ELISA assay to examine the expression level of TNF‐α and IL‐6 in liver tissues (Figure 3A). The correlation analysis demonstrated that the concentrations of TNF‐α (*r* = 0.559, *P* = .005) and IL‐6 (*r* = 0.643, *P* = .001) were positively associated with bioluminescence intensity in the mice in the liver injury groups (G1, G2 and G3) (Figure 3B), which implied that the hepatic inflammatory cytokines up‐regulated by liver injury accelerated HCC progression in vivo. Previous studies demonstrated that the STAT3 activation promoted the tumour progression by epigenetic modification of epithelial‐to‐mesenchymal transition (EMT) phenotypes.^27^, ^28^ Thus, we examined the STAT3/EMT pathway in the normal liver group and liver injury groups (G0‐G3), and found that chronic liver injury increased the expression level of p‐STAT3, E‐cadherin and Vimentin in HCC tissues (Figure 3C). Importantly, the higher level of p‐STAT3, E‐cadherin and Vimentin were found in liver injury groups for 6 and 10 weeks compared to 4 weeks (G2/G3 vs G1; Figure 3H), indicating that the increased levels of inflammatory factors (TNF‐α and IL‐6) in liver tissues were concordant with the activation extent of STAT3/EMT signalling pathway in HCC (Figure 3A,C).

We further investigated the effects of exogenous TNF‐α and IL‐6 on HCC proliferation and invasion in vitro. The CCK8 and colony formation assays showed that each cytokine (TNF‐α or IL‐6) alone significantly improved the proliferation of HCC cells. Interestingly, when the two agents are used together, they produce much stronger enhancing effect on proliferation (Figure 3D). In addition, TNF‐α or IL‐6 individually promoted the migration and invasion, whereas their combination resulted in more significant improvement in HCC cells (Figure 3E). STAT3/EMT pathway was detected in HCC cells after treatment with exogenous TNF‐α or/and IL‐6. The results showed that each agent alone clearly promoted the activation of STAT3 (Figure 3F) and induced a typical change of EMT markers (down‐regulation of the epithelial marker E‐cadherin and up‐regulation of the mesenchymal markers Vimentin and N‐cadherin) (Figure 3F,G). Moreover, combination treatment exerted a stronger effect on the STAT3 activation and EMT change (Figure 3F,G). The above results indicated the tumour‐promoting effect of chronic liver inflammation by multiple cytokines.

**FIGURE 4:**
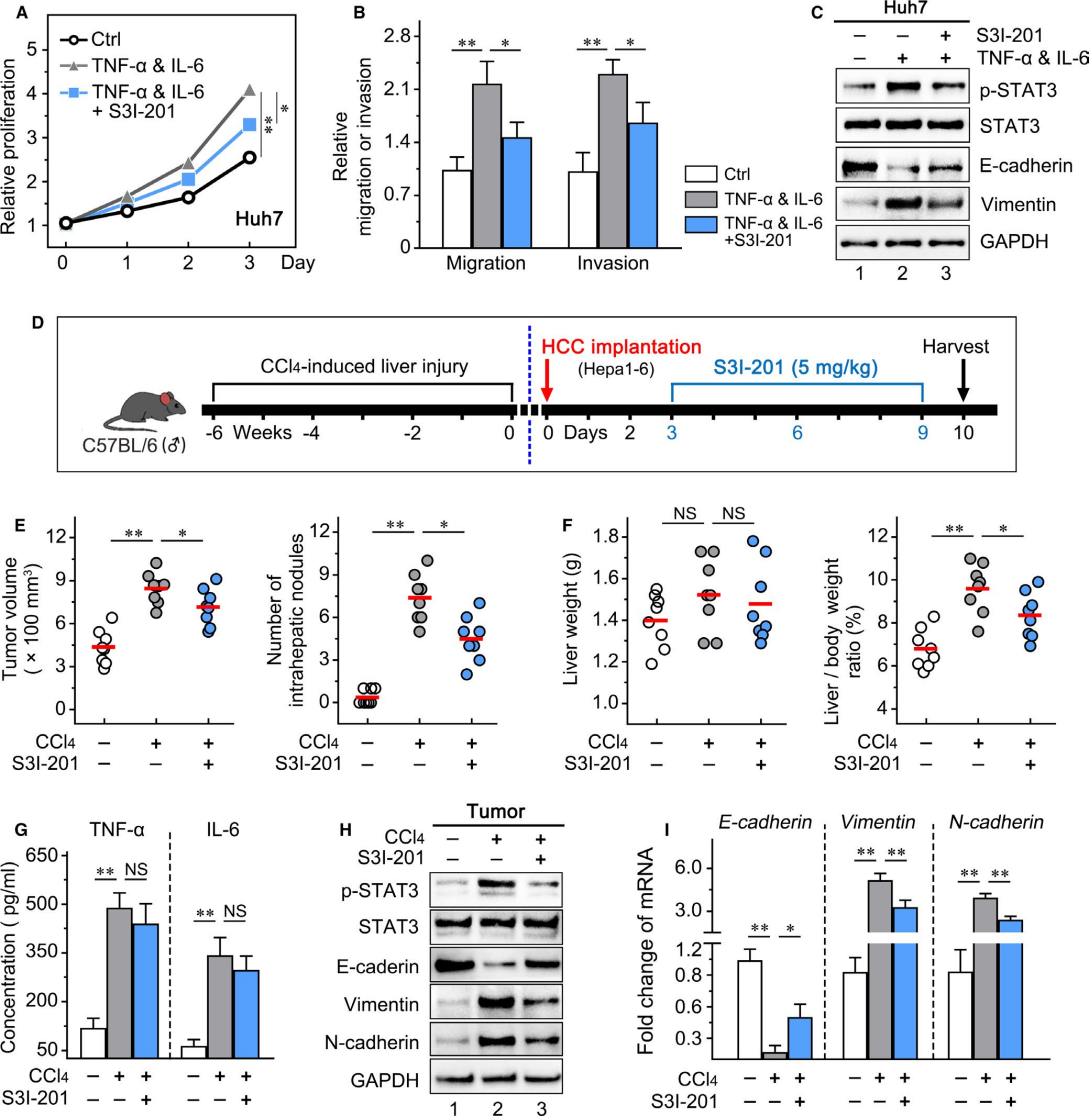
STAT3 inhibitor S3I‐201 attenuates the HCC growth and metastasis exacerbated by the hepatic inflammatory microenvironment. Huh7 cells were pre‐treated by DMSO (Ctrl), TNF‐α and IL‐6, and TNF‐α and IL‐6 with S3I‐201, (A) CCK8 assays to detect the growth inhibition for each group; (B) transwell migration assay to show the S3I‐201 therapy on mobility of Huh7 cells; (C) The effect of S3I‐201 on STAT3, p‐STAT3 and EMT‐related markers in Huh7. D, Mice were gavaged by olive oil (normal liver group) or CCl_4_ for 6 wk (liver injury group) and then orthotopically implanted with Hepa1‐6 cells. Among the liver injury group, part of the mice (treatment group) were treated with S3I‐201 by intraperitoneal injection from day 3 to day 9 after implantation. All mice were killed at 10 d after implantation. Statistical analysis of (E) tumour volume, number of intrahepatic nodules, (F) liver weight and the liver/body weight ratio in three groups at the end point. G, The levels of TNF‐α and IL‐6 in liver tissue were examined by ELISA. H, The effect of S3I‐201 on the STAT3/EMT pathway in tumour tissues was detected by Western blot. I, The mRNA level of EMT‐related markers in tumour tissues was confirmed by RT‐PCR. Data presented are means ± SD. NS, not significant. *P < .05; **P < .01

To further verify the role of STAT3 activation in HCC induced by hepatic inflammatory cytokines, the STAT3 inhibitor S3I‐201 was applied in vitro and in vivo. We found that S3I‐201 significantly reduced HCC proliferation and invasion enhanced by combination treatment of TNF‐α and IL‐6 (Figure 4A,B). Meanwhile, the increased expression of p‐STAT3, E‐cadherin and Vimentin in HCC cells was reduced by S3I‐201 (Figure 4C). The orthotopic HCC mouse model with chronic liver injury was administered S3I‐201 (Figure 4D). We found that the accelerated tumour growth (*P* = .045) and intrahepatic metastasis by hepatic inflammatory microenvironment (*P* = .003; Figure 4E) were effectively delayed by S3I‐201. Consistently, the increased liver/body weight ratio of mice in the liver injury group was clearly inhibited by S3I‐201 (*P* = .040; Figure 4F). Moreover, although it did not significantly affect the level of TNF‐α and IL‐6 in liver tissues (Figure 4G), S3I‐201 obviously restrained the enhanced STAT3 activation and EMT change of tumour tissues in the liver injury group (Figure 4H,I). These data indicated that liver inflammation enhanced HCC growth and intrahepatic metastasis by the pro‐inflammatory cytokines‐induced activation of STAT3.

### 3.4 Hepatic inflammatory microenvironment drives acquired sorafenib resistance via the STAT3 activation of HCC

**FIGURE 5:**
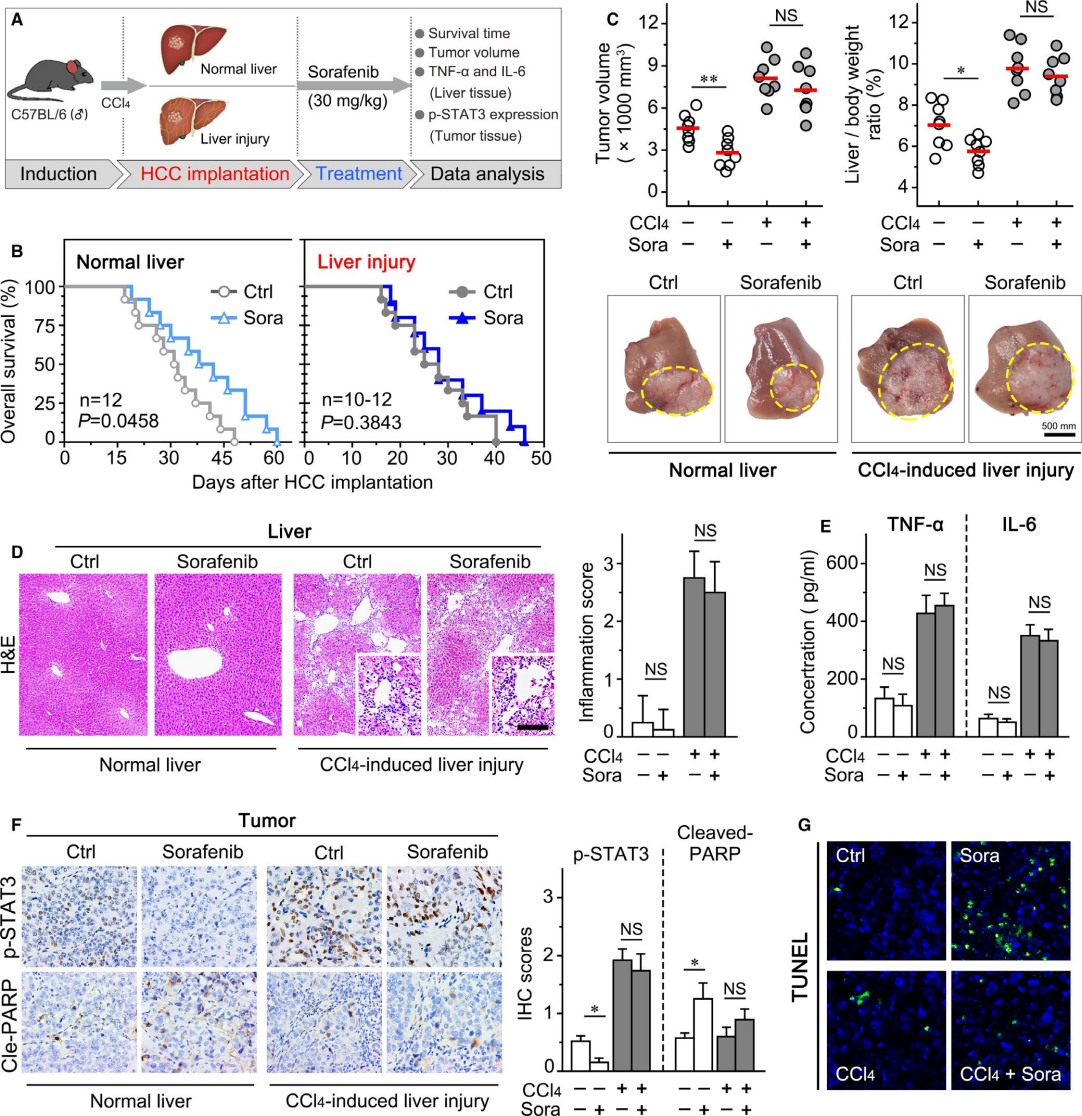
Hepatic inflammatory microenvironment impairs the sorafenib efficacy in the orthotopic mouse model of HCC. A, Experimental design to investigate the effects of hepatic inflammatory microenvironment on sorafenib resistance in the orthotopic HCC mouse model. The mice were divided into four groups: vehicle control (normal liver), sorafenib (normal liver), vehicle control (chronic liver injury) and sorafenib (chronic liver injury). B, Kaplan‐Meier survival analysis of the mice continuously administered with CCl_4_ gavage and sorafenib after HCC implantation to the humane end point (n ≥ 10/group). C, Part of the mice in the four groups were killed for assessment of tumour burden (tumour volume and the liver/body weight ratio) after sorafenib treatment for 7 d (n = 8/group; upper panel); the representative images of tumour morphology at the end point (lower panel). The inflammation and level of TNF‐α and IL‐6 in mouse liver tissues were examined by H&E staining (D) and ELISA (E), respectively. F, Representative images of IHC staining with p‐STAT3 and cleaved‐PARP antibody on tumour tissues from four groups (Left panel); statistical analysis of their IHC score (Right panel). G, Representative images of in situ apoptotic cells in tumour tissues detected by TUNEL assay. Data presented are means ± SD. NS, not significant. *P < .05; **P < .01

By examination of the phase 3 trials (SHARP and ORIENTAL), the recent study found that inflammation is an adverse prognostic factor for HCC patients with sorafenib treatment.^29^ We next investigated the effect of hepatic inflammatory microenvironment on sorafenib resistance in vivo (Figure 5A). The survival analysis indicated that sorafenib significantly prolonged the median survival time in normal liver mice (31.0 ± 3.5 vs 38.0 ± 6.1 days; *P* = .046), but did not prolong the survival time in these liver injury mice (25.0 ± 4.3 vs 28.0 ± 2.3 days; *P* = .384; Figure 5B). In addition, sorafenib effectively decreased the tumour volume and liver/body weight ratio in the normal liver group but not in the liver injury group (Figure 5C). Furthermore, we used H&E staining to detect the massive infiltration of periportal inflammatory cell and confirmed the remarked hepatic inflammation elicited by liver injury (Figure 5D). However, sorafenib did not significantly affect the degree of liver inflammation and the levels of TNF‐α and IL‐6 in the liver injury group (Figure 5D,E).

In addition, we found that sorafenib significantly reduced the levels of p‐STAT3 (Figure 5F), and induced the distinct apoptosis of HCC in normal liver group which manifested by the increased level of cleaved PARP (Figure 5F) and the numbers of apoptotic cells (Figure 5G). However, the excessive STAT3 activation within the hepatic inflammatory microenvironment was unable to be inhibited by sorafenib (Figure 5F). The level of cleaved PARP and the apoptosis of HCC were not effectively impacted by sorafenib, demonstrating that the sorafenib‐induced apoptosis was diminished in the liver injury group (Figure 5F,G). These data suggested that chronic liver inflammation may account for the sorafenib resistance.

**FIGURE 6:**
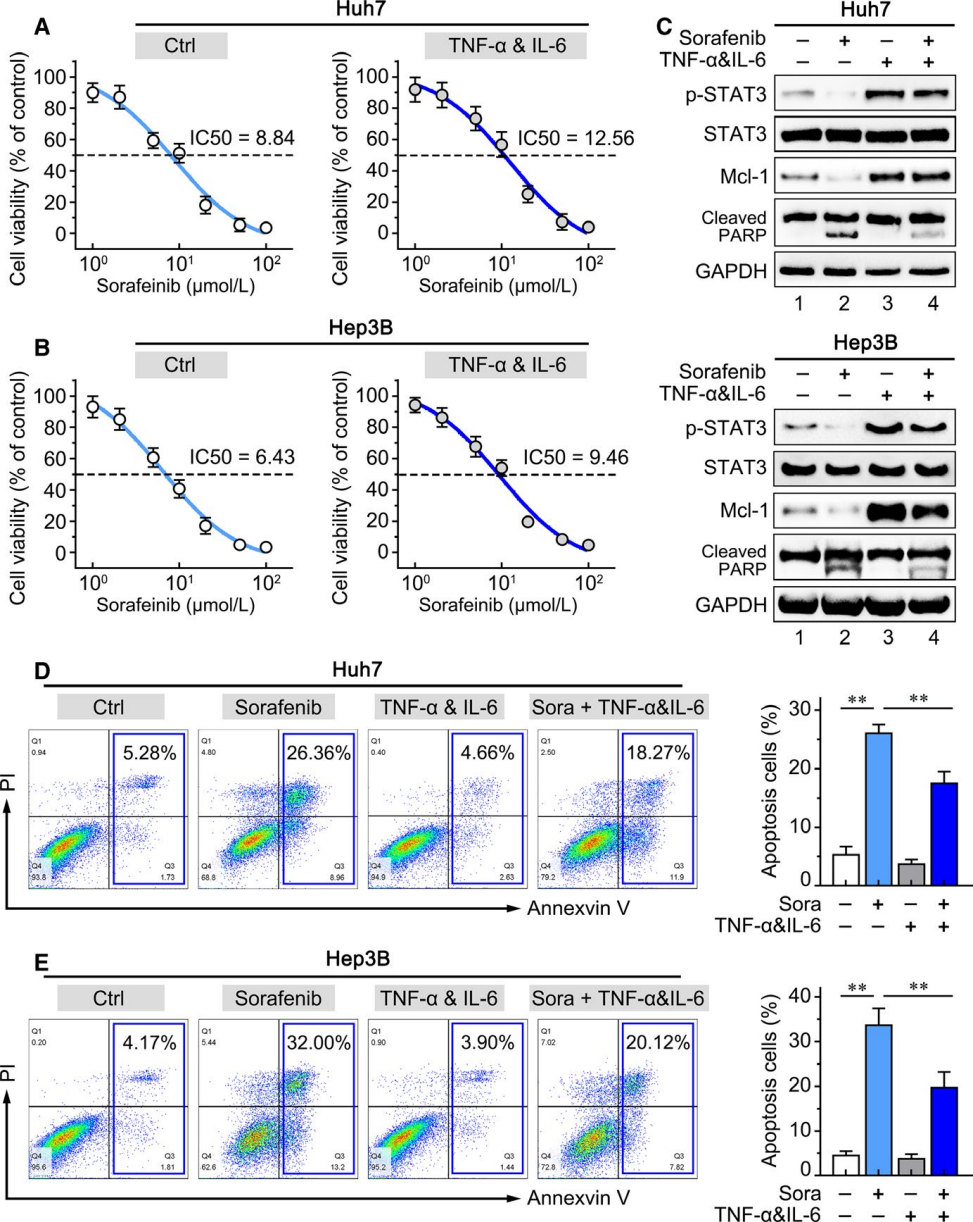
Exogenous inflammatory cytokines derive the sorafenib resistance via activation of STAT3. Dose‐dependent effects of sorafenib on the viability of (A) Huh7 and (B) Hep3B cells with or without TNF‐α and IL‐6 stimulation. HCC cells were treated with sorafenib (5 μmol/L) in the absence or presence of TNF‐α and IL‐6, (C) the protein levels of p‐STAT3, STAT3, Mcl‐1 and cleaved‐PARP were measured by Western blotting, (D) Huh7 and (E) Hep3B cells were stained with FITC‐conjugated Annexin V and PI, and then analysed by flow cytometry (Left panel). Histogram analysis of apoptotic ratio (Right panel). Data presented are means ± SD. NS, not significant. *P < .05; **P < .01

Further, we treated HCC cells by different doses of sorafenib with or without hepatic inflammatory cytokines (TNF‐α and IL‐6). With additional treatment of TNF‐α and IL‐6, the IC_50_ values of the Huh7 and Hep3B cells were elevated from 8.84 to 12.56 μmol/L and 6.43 to 9.46 μmol/L, respectively (Figure 6A,B). Moreover, the level of p‐STAT3 and the anti‐apoptosis protein Mcl‐1 mediated by STAT3 were decreased by sorafenib. However, the inhibitory effects of sorafenib on p‐STAT3 and Mcl‐1 were abolished when HCC cells pre‐treated with exogenous inflammatory cytokines (Figure 6C). In addition, the cleaved PARP expression (Figure 6C) and the apoptotic HCC cells (Figure 6D,E) induced by sorafenib were down‐regulated by exogenous TNF‐α and IL‐6. The results suggested that hepatic inflammatory microenvironment impaired the anti‐tumour effect of sorafenib by cytokines‐induced activation of STAT3.

### 3.5 STAT3 antagonist reverses the inflammation‐induced HCC resistance to sorafenib

**FIGURE 7:**
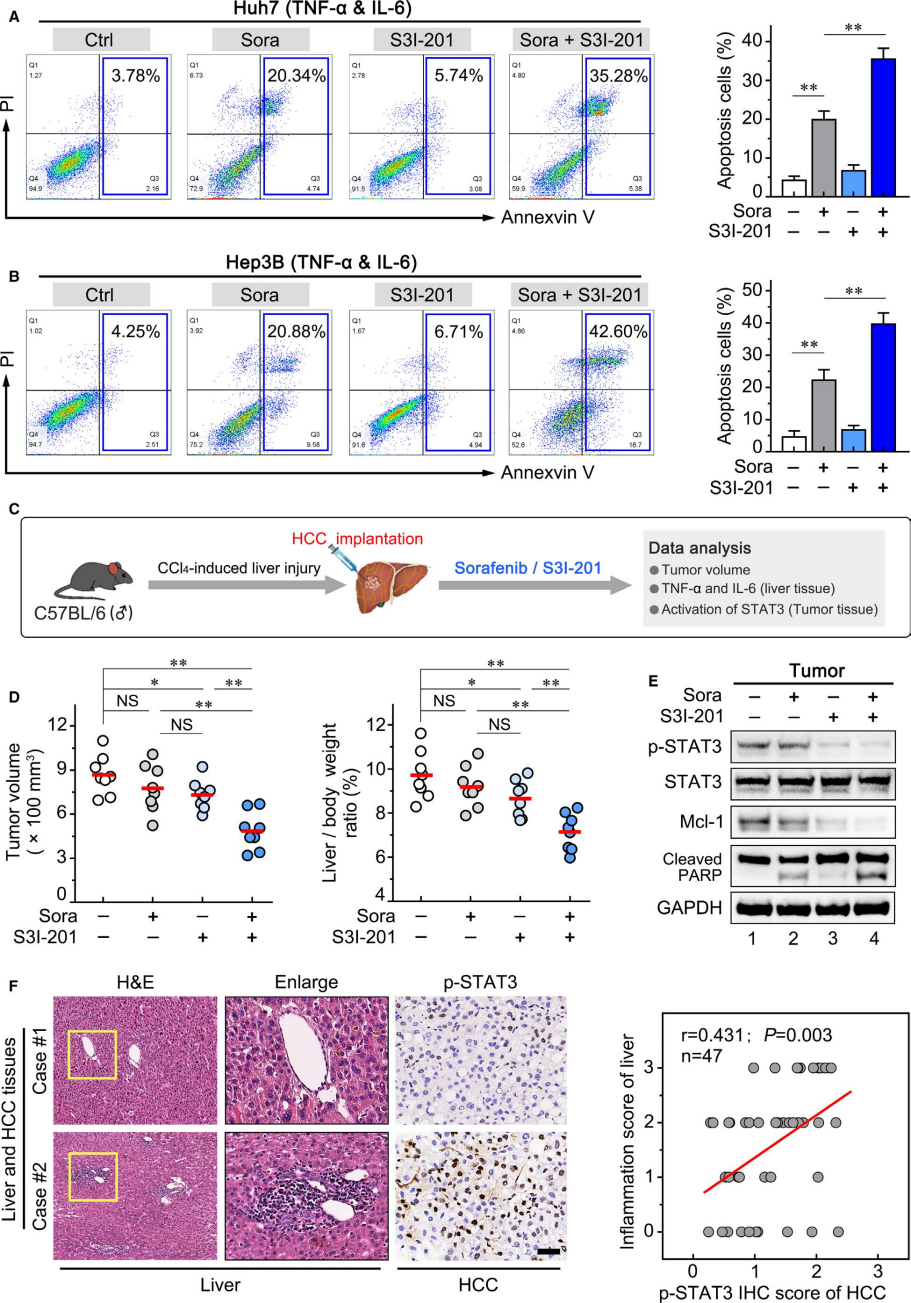
S3I‐201 improves the sorafenib efficacy in vitro and in vivo. After treatment with sorafenib (5 μmol/L) and S3I‐201 (100 μmol/L) in the presence of exogenous TNF‐α and IL‐6, (A) Huh7 and (B) Hep3B cells were stained with FITC‐conjugated Annexin V and PI (Left panel), and monitored using flow cytometry (Left panel). Histogram analysis of apoptotic ratio (Right panel). C, Experimental design of combination therapy in vivo. All mice were administered CCl_4_ for 6 wk and then orthotopically implanted with Hepa1‐6 cells. These mice were further divided into four groups (n = 8/group): control group, sorafenib group, S3I‐201 group and combination therapy group. Sorafenib and S3I‐201 were administered from day 3 to day 9 after implantation. All mice were killed at 10 d after implantation. D, Tumour volume per liver (100 mm^3^) and liver/body weight ratio (%) were calculated in four groups. E, STAT3‐related proteins (p‐STAT3, total STAT3 and Mcl‐1) and cleaved PARP were detected by Western blotting. 47 pairs of HCCs and non‐tumour tissues were collected, (F) the representative liver tissue stained by H&E from HCC patients with mild and severe chronic liver inflammation (case 1 and case 2), and the corresponding tumour tissue stained for p‐STAT3 was shown (left panel), p‐STAT3 expression in human HCC tissues was increased as the inflammation increased in liver tissues (right panel). Data presented are means ± SD. NS, not significant. *P < .05; **P < .01

To determine the effect of STAT3 activation in sorafenib resistance elicited by hepatic inflammatory microenvironment, we used STAT3 antagonist (S3I‐201) combined with sorafenib to treat in vitro and in vivo. In the presence of inflammatory cytokines (TNF‐α and IL‐6), we found that sorafenib had not significant effect on STAT3 activation, while S3I‐201 effectively inhibited the p‐STAT3 expression in Huh7 and Hep3B (Figure S1). Furthermore, S3I‐201 dramatically enhanced the ability of sorafenib to induce cell death (Figure 7A,B). In an orthotopic HCC mouse model with chronic liver injury (Figure 7C), S3I‐201 combined with sorafenib greatly reduced tumour volume (*P* = .001), liver/body weight ratio (*P* < .001) and intrahepatic metastasis (*P* < .001) compared to sorafenib alone (Figure 7D, Figure S2). The ELISA assays indicated that the level of TNF‐α and IL‐6 in liver tissues did not decrease by treatment together or alone (data not shown). Importantly, S3I‐201 down‐regulated the expression of p‐STAT3 and Mcl‐1 in HCC tissues and contributed to the activation of PARP cleavage induced by sorafenib (Figure 7E). These data revealed that STAT3 inhibitor contributed to sorafenib sensitivity of HCC through enhancing apoptosis.

To examine the relationship between the status of STAT3 activation in human HCC and liver inflammation, 47 pairs of HCCs and the non‐tumour part in fibrotic liver were stained for p‐STAT3 and liver inflammation, respectively. Among all the tumour samples examined, positive p‐STAT3 staining was identified in 36 tumour samples (76.5%). Moreover, correlation analysis demonstrated that the intensity of p‐STAT3 in HCC tissue was positively correlated with the degree of liver inflammation (*r* = 0.431, *P = *.003; Figure 7F). The results indicated that the increase of liver inflammation was accompanied with STAT3 activation in HCC patients.

## 4 DISCUSSION

Most of HCC occurs in the setting of liver fibrosis, underlying the important roles of the pro‐inflammatory and pro‐fibrotic microenvironment in the fibrotic liver for HCC development.^12^ However, due to a paucity of suitable in vivo models, the effects and mechanisms of the hepatic pathologic microenvironment on intrahepatic HCC progression and response to systematic therapy remain largely unexplored.^10^, ^11^ In the present study, a syngeneic orthotopic HCC mouse model with chronic liver injury was established to mimic the intrahepatic growth and metastasis process. We demonstrated that chronic liver inflammation accelerated the intrahepatic HCC progression and impaired the sorafenib efficacy via cytokines‐induced STAT3 activation.

The role of inflammation in tumour progression remains controversial. Some studies indicated that inflammation facilitates the tumour growth and metastasis by inducing the effector molecules,^30^ disabling the function of tumour‐specific T cells and enhancing the conversion of progenitor cells to cancer stem cells.^31^, ^32^ In contrast, other studies indicated that inflammation up‐regulated nitrite and TNF‐α activating caspase‐3 induced apoptosis in the tumour cells,^33^ and enhanced antitumour T cell immunity by decreasing immunosuppressive infiltration of myeloid‐derived suppressor cells and Treg cells.^34^ In addition, the dual role of inflammation in tumour were also demonstrated,^35^ suggesting that anti‐tumour and pro‐tumour inflammatory mechanisms co‐existed in the process of tumour progression.^13^ Due to the intense connection between HCC and organ inflammation, preclinical studies are indeed in much need to verify the effect of liver inflammatory microenvironment on HCC progression. Our study developed a preclinical mouse models of orthotopic HCC and provided the evidences that pro‐tumour effect of liver inflammatory microenvironment dominated in the HCC progression.

The non‐resolving inflammation involved in tumour initiation and progression were elicited by two distinct pathway. The intrinsic pathway is induced by alterations in tumour‐associated genetic factors. The extrinsic pathway driven by exogenous factors (infection, obesity or environmental factors) establishes an organ inflammatory condition. Notably, as it usually develops in the chronic liver injury, HCC has a more intense connection with organ inflammation induced by an extrinsic pathway than other cancers.^11^, ^15^ In addition, previous studies have indicated that hepatic inflammatory genes were identified as distinct independent prognostic factors for HCC patients.^36^, ^37^ Here, although further research is needed to deeply explore the tumour‐promoting mechanism of the hepatic inflammatory microenvironment, we provided direct evidence for the role of chronic liver inflammation on HCC progression. Besides, emerging evidence suggested that hepatic pathologic microenvironment drives aggressive HCC growth via a profound immunosuppressive mechanism.^22^ In view of the important role of the liver microenvironment in HCC progression, and the advantage of altering the microenvironment as treatment for tumour that are less likely to develop resistance,^38^ modulation of hepatic pathologic microenvironment may contribute to develop the novel therapeutic approaches for HCC patients.^39^

Several studies have shown that tumours with STAT3 activation become more aggressive and are associated with poor prognosis in HCC patients.^40^ Until now, the inducers for STAT3 activation in HCC have not been fully understood. It is generally considered that STAT3 is activated by cytokines and growth factors produced within the tumour microenvironment.^11^ Our results suggested that hepatic inflammatory microenvironment also activated the STAT3 of the HCC in a paracrine manner, providing new insight regarding STAT3 activation in human HCC. Increasing evidence demonstrates that STAT3 activation results in sorafenib resistance,^18^ while other studies implied that sorafenib could also inhibit the HCC progression by reducing STAT3 activation.^41^, ^42^ In the present study, the relationship between sorafenib treatment and STAT3 activation was clarified. Indeed, the STAT3 activation could be inhibited by sorafenib in an HCC mouse model with normal liver. However, the hepatic inflammatory microenvironment was able to constitutively activate STAT3 of HCC, impairing the sorafenib efficacy. We found that STAT3 inhibitor S3I‐201 did not directly induce the HCC cell death and had negligible effect on the expression of cleaved PARP. These findings were consistence with the other study.^18^ However, the combination treatment of S3I‐201 and sorafenib significantly increased HCC apoptosis and cleaved PARP level. Previous studies demonstrated that STAT3 promoted the treatment resistance through reducing cleaved PARP level via regulating the downstream protein such as Mcl‐1, survivin and cyclin D1.^43^ As the member of Bcl‐2 family and an important downstream effectors, Mcl‐1 has been reported to enhance the maintenance of mitochondrial membrane permeability by inhibiting pro‐apoptotic effect and Apaf‐1,^44^ and subsequently decreased the drug‐induced cleaved PARP.^45^ Our results demonstrated that STAT3 inhibitor S3I‐201 promoted the sorafenib‐induced apoptosis and expression of cleaved PARP in HCC by down‐regulating Mcl‐1. Considering that the increase of liver inflammation was accompanied with p‐STAT3 up‐regulation of tumour tissues for HCC patients (Figure 7F), STAT3 inhibitor may improve the sorafenib efficacy for HCC patients with marked liver inflammation.

In summary, we demonstrated that inflammatory microenvironment of fibrotic liver elicited by hepatic injury promotes HCC progression and sorafenib resistance by cytokines‐induced activation of STAT3. Targeting STAT3 of HCC has a potential clinical value to improve the sorafenib efficacy in HCC patients with chronic liver inflammation.

## CONFLICT OF INTEREST

The authors do not have any disclosures to report.

## AUTHOR CONTRIBUTIONS

**Yuchuan Jiang:** Conceptualization (equal); data curation (equal); formal analysis (equal); funding acquisition (equal); investigation (equal); methodology (equal); project administration (equal); resources (equal); software (equal); supervision (equal); validation (equal); visualization (equal); writing‐original draft (equal); writing‐review and editing (equal). **Peng Chen:** Conceptualization (equal); data curation (equal); formal analysis (equal); funding acquisition (equal); resources (equal); software (equal); supervision (equal); validation (equal); visualization (equal). **Kaishun Hu:** Funding acquisition (equal); investigation (equal); methodology (equal); writing‐original draft (equal); writing‐review and editing (equal). **Guanqi Dai:** Formal analysis (equal); software (equal); supervision (equal). **Jinying Li:** Data curation (equal); investigation (equal); visualization (equal). **Dandan Zheng:** Writing‐original draft (equal); writing‐review and editing (equal). **Hui Yuan:** Software (equal). **Lu He:** Validation (equal). **Penghui Xie:** Data curation (equal). **Mengxian Tu:** Resources (equal). **Shuang Peng:** Methodology (equal). **Chen Qu:** Software (equal). **Wenyu Lin:** Writing‐original draft (equal). **Raymond Chung:** Writing‐review and editing (equal). **Jian Hong:** Conceptualization (equal); data curation (equal); funding acquisition (equal); investigation (equal); methodology (equal); project administration (equal); resources (equal); validation (equal); visualization (equal); writing‐original draft (equal); writing‐review and editing (equal).

## Supporting information

Fig S1‐S2

Supplementary Material

## Data Availability

The data that support the findings of this study are openly available (in figshare at http://doi.org/[doi]). And all data generated or analyzed during this study are included in this article.
