# Supplementary material for: Inflammatory microenvironment of fibrotic liver promotes hepatocellular carcinoma growth, metastasis and sorafenib resistance through STAT3 activation
Source: J Cell Mol Med. 2021 Jan 7;25(3):1568–82. doi: 10.1111/jcmm.16256 (PMC7875922; doi:10.1111/jcmm.16256)
Supplement: Supplementary file 1 — Fig S1‐S2 [file JCMM-25-1568-s001.docx]

**Supplementary Information**

**Inflammatory microenvironment of fibrotic liver promotes hepatocellular carcinoma growth, metastasis and sorafenib resistance** **through STAT3 activation**

Yuchuan Jiang^1^, Peng Chen^1^, Kaishun Hu^2^, Guanqi Dai^1^, Jinying Li^3^, Dandan Zheng^1^, Hui Yuan^1^, Lu He^4^, Penghui Xie^1^, Mengxian Tu^1^, Shuang Peng^5^, Chen Qu^1^, Wenyu Lin^6^, Raymond T Chung^6^, and Jian Hong^1^

*^1^Department of Abdominal Surgery, Integrated Hospital of Traditional Chinese Medicine, Southern Medical University, Guangzhou, Guangdong 510315, China*

*^2^Guangdong Provincial Key Laboratory of Malignant Tumor Epigenetics and Gene Regulation, Medical Research Center, Sun Yat-Sen Memorial Hospital, Sun Yat-Sen University, Guangzhou, Guangdong 510120, China*

*^3^Department of Gastroenterology, Guangzhou Overseas Chinese Hospital, Jinan University, Guangzhou, Guangdong 510632, China*

*^4^Department of Radiotherapy, Affiliated Cancer Hospital & Institute of Guangzhou Medical University, Guangzhou, Guangdong 510095, China*

*^5^Department of Pathophysiology, School of Medicine, Jinan University, Guangzhou, Guangdong 510632, China.*

*^6^Liver Center and Gastrointestinal Division, Massachusetts General Hospital, Harvard Medical School, Boston, MA 02114, USA*

**Correspondence:**

Dr. Jian Hong, Department of Abdominal Surgery, Integrated Hospital of Traditional Chinese Medicine, Southern Medical University, Guangzhou, Guangdong 510315, China; Phone & Fax: (+86 20) 6165 0514; E-mail: [Hongjian7@hotmail.com](mailto:Hongjian7@hotmail.com).


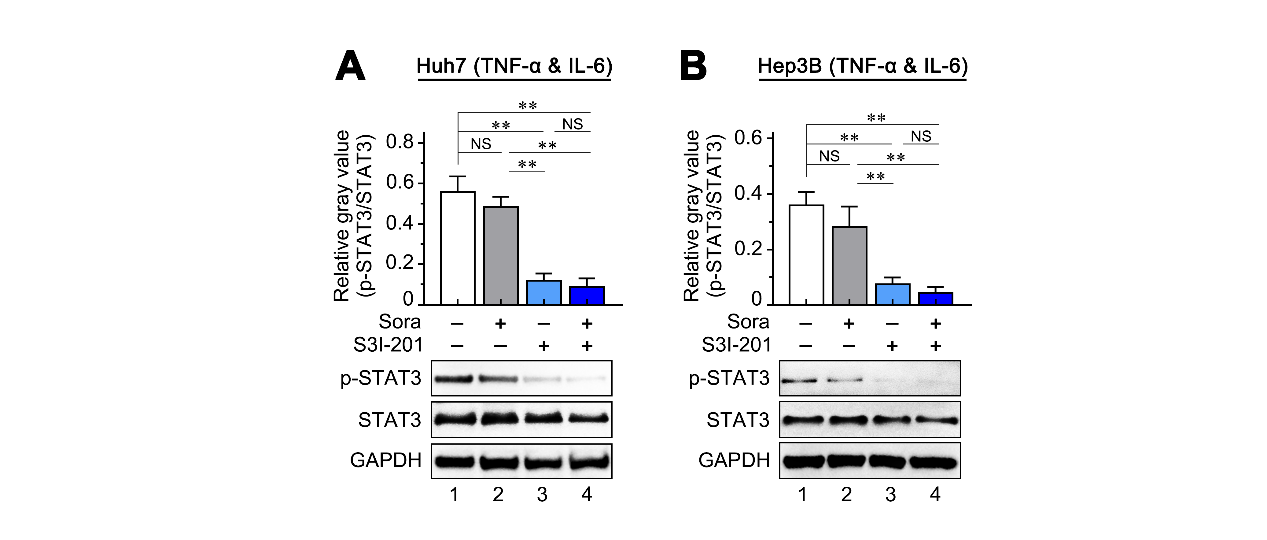


**Supplementary Figure 1. S3I-201 exerted the stronger inhibitory effect on STAT3 compared to sorafenib *in vitro* under TNF-α and IL-6 stimulation.** After treatment with sorafenib (5 μmol/L) and S3I-201 (100 μmol/L) in the presence of exogenous TNF-α and IL-6 for 24 h, Huh7 (**A**) and Hep3B (**B**) cells were collected and examined the protein levels of p-STAT3 and STAT3 by western blotting, the gray value based on the expression of p-STAT3 and STAT3 were analyzed.


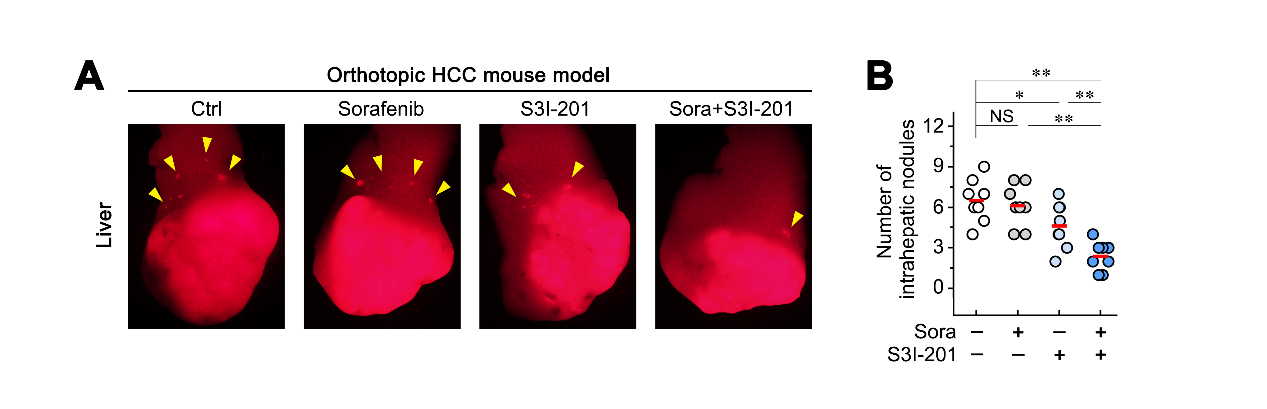


**Supplementary Figure 2. Combination treatment of S3I-201 and sorafenib effectively inhibited the intrahepatic metastasis *in vivo***. Experimental design of combination therapy as showed in Figure 7C: these orthotopic HCC mouse model with hepatic inflammatory microenvironment were divided into four groups (n = 8 mice/group): control, sorafenib treatment, S3I-201 treatment, and combination therapy of S3I-201 and sorafenib. The number of intrahepatic metastasis were calculated and compared by fluorescence and the arrow indicated the intrahepatic nodules (**A**), the statistical analysis of the number of intrahepatic nodules in four groups (**B**).
