# Supplementary material for: Inflammatory microenvironment of fibrotic liver promotes hepatocellular carcinoma growth, metastasis and sorafenib resistance through STAT3 activation
Source: J Cell Mol Med. 2021 Jan 7;25(3):1568–82. doi: 10.1111/jcmm.16256 (PMC7875922; doi:10.1111/jcmm.16256)
Supplement: Supplementary file 2 — Supplementary Material [file JCMM-25-1568-s002.docx]

**Supplementary Information**

**Inflammatory microenvironment of fibrotic liver promotes hepatocellular carcinoma growth, metastasis and sorafenib resistance** **through STAT3 activation**

Yuchuan Jiang^1^, Peng Chen^1^, Kaishun Hu^2^, Guanqi Dai^1^, Jinying Li^3^, Dandan Zheng^1^, Hui Yuan^1^, Lu He^4^, Penghui Xie^1^, Mengxian Tu^1^, Shuang Peng^5^, Chen Qu^1^, Wenyu Lin^6^, Raymond T Chung^6^, and Jian Hong^1^

*^1^Department of Abdominal Surgery, Integrated Hospital of Traditional Chinese Medicine, Southern Medical University, Guangzhou, Guangdong 510315, China*

*^2^Guangdong Provincial Key Laboratory of Malignant Tumor Epigenetics and Gene Regulation, Medical Research Center, Sun Yat-Sen Memorial Hospital, Sun Yat-Sen University, Guangzhou, Guangdong 510120, China*

*^3^Department of Gastroenterology, Guangzhou Overseas Chinese Hospital, Jinan University, Guangzhou, Guangdong 510632, China*

*^4^Department of Radiotherapy, Affiliated Cancer Hospital & Institute of Guangzhou Medical University, Guangzhou, Guangdong 510095, China*

*^5^Department of Pathophysiology, School of Medicine, Jinan University, Guangzhou, Guangdong 510632, China.*

*^6^Liver Center and Gastrointestinal Division, Massachusetts General Hospital, Harvard Medical School, Boston, MA 02114, USA*

**Correspondence:**

Dr. Jian Hong, Department of Abdominal Surgery, Integrated Hospital of Traditional Chinese Medicine, Southern Medical University, Guangzhou, Guangdong 510315, China; Phone & Fax: (+86 20) 6165 0514; E-mail: Hongjian7@hotmail.com.

**Supplementary Materials and Methods**

**Flow cytometry**

Fresh mouse liver tissues were finely chopped and dissociated into single-cell suspensions (5). After removal of red blood cells and liver cells, the leukocytes were further purified by the magnetic-activated cell sorting separator with CD45 magnetic beads (Miltenyi Biotec, CA, USA). After incubating with BV711-labeled F4/80 (BD Biosciences, CA, USA) and BV510-labeled CD11b (BD Biosciences), liver-infiltrated inflammatory macrophages were detected by flow cytometer (BD LSRFortessa X-20).

**Quantitative real-time PCR**

Total RNA was isolated from the indicated tissue and cells by using TRIzol reagent (Invitrogen, CA, USA) and reverse transcribed into cDNA. Quantitative RT-PCR (qRT-PCR) was performed using the SYBR Green Master Mix (Takara, Kyoto, Japan). The amplification conditions were: 94°C 3 min; 40 cycles of 95°C 20 s, 60°C 40 s and 72°C 20 s; and elongation at 72°C for 5 min. The mRNA expression level of target genes was normalized to GAPDH by using the 2-ΔΔCt method. Primer sequences are available in Supplementary table 1.

**Enzyme-Linked Immunosorbent Assay (ELISA)**

The liver tissues collected above were weighed and homogenized at 4°C. Liver homogenates were centrifuged at 14000xg for 10 min at 4°C. Supernatants were transferred to clean microcentrifuge tubes for detection. Specific ELISA kits (Jiangsu Meimian industrial, Jiangsu, China) were used to quantitate TNF-α and IL-6 in mouse liver tissue according to the manufacturer’s instructions.

**Cell proliferation assay**

Huh7 cells were seeded at 1,000 cells per well in 96-well microplates and incubated in normal growth medium for 24 h. Subsequently, the cells were treated with DMSO, TNF-α (40 ng/ml), IL-6 (20 ng/ml), TNF-α and IL-6, and TNF-α and IL-6 with S3I-201 for an additional 24, 48, or 72 h. In another assay, Huh7 and Hep3B were seeded at 1,000 cells per well in 96-well microplates and treated with varying concentrations of sorafenib with or without TNF-α and IL-6 for 48 h. Cell viability was measured using the Cell Counting Assay Kit-8 (CCK-8; Dojindo, Kumamoto, Japan) according to the manufacturer’s instructions.

**Colony formation assay**

Cells were seeded onto 6-well plates (500 cells/well) and cultured at 37°C with 5% CO2 overnight. The cells were then treated by DMSO, TNF-α, IL-6, TNF-α & IL-6, and TNF-α & IL-6 with S3I-201 for about 2 weeks until colonies were visible. The cells were washed with PBS and new complete medium containing the above mentioned agents was replaced every 3 days. The colonies were washed with PBS twice, fixed with 4% paraformaldehyde for 20 min, and then washed with PBS followed by staining with 0.1% crystal violet solution for 15 min. the number of colonies was counted under the microscope. Three different independent experiments were performed.

**Transwell migration and invasion assays**

Cell migration and invasion assays were performed on transwell chambers with 8-μm pore-size filters without (for migration) or with (for invasion) coated Matrigel (BD Biosciences). Cells were trypsinized and resuspended in serum-free medium and either DMSO, TNF-α, IL-6, TNF-α and IL-6, or TNF-α and IL-6 with S3I-201. 250 μl of cell suspension (1 x 10^5^ cells) was added to the upper chambers in a transwell insert, and the upper chambers were then placed into the wells of a 24-well plate. 750 μl culture medium containing 20% fetal bovine serum (FBS) was added to the lower chamber. After transwell inserts were cultured at 5% CO2 at 37°C for 24 h, cells on the top of the membrane were removed with a cotton swabs. Cells attached on the underside of the membrane were fixed and stained with 0.1% crystal violet. After washing with phosphate-buffered saline (PBS), the number of cells was counted in three random microscopic fields under the microscope.

**Western blotting**

The total cellular protein and tissue protein was extracted by RIPA Lysis Buffer (Thermo Fisher Scientific, MA, USA) and RIPA Lysis Buffer (Thermo Fisher Scientific) containing protease inhibitors and phosphatase inhibitors (Thermo Fisher Scientific). The protein concentrations of the cell lysates were measured using a Pierce^TM^ BCA Protein Assay Kit (Thermo Fisher Scientific) and equalized before loading. Equal amount of protein extracts from HCC cells or tissues were separated by SDS–PAGE, and transferred onto polyvinylidene fluoride membranes (Sigma-Aldrich, MO, USA). Immunoblot analyses were carried out using the appropriate antibodies, and the bands were visualized using an SuperSignal^TM^ West Pico PLUS chemiluminescence Substrate (Thermo Fisher Scientific).

**Immunofluorescence assay**

Huh7 cells (2 × 10^3^/well) were seeded in 24-well plates, incubated for 24 h, then treated with dimethyl sulfoxide (DMOS), TNF-α (40 ng/ml), IL-6 (20 ng/ml) or TNF-α and IL-6. Next, HCC cells were washed and fixed with 4% paraformaldehyde (PFA) for 20 min, and permeabilized in 0.3% Triton X-100. Incubation with monoclonal rabbit anti- p-STAT3 antibody overnight at 4 °C was followed by incubation with fluorescein isothiocyanate (Alexa Fluor® 555)-labeled goat anti-rabbit IgG secondary antibody for 60 min in a dark wet box. Following triplicate washes with (PBS with tween) PBS-T, the cells were counterstained with 4′, 6-diamidino-2-phenylindole (DAPI, CWBIO) for 5 min. The results were photographed under confocal laser scanning microscopy.

**Histological and immunohistological analysis of liver sections**

Liver and tumor tissues were fixed with 10% formalin, embedded in paraffin and cut into 2 mm sections for staining with hematoxylin-eosin (H&E), Sirius red and immunohistochemistry according to standard procedures. The amount of Sirius red staining was quantified with ImageJ (ImageJ, http://imagej.net/). Fibrosis was scored according to the METAVIR scoring system (1). Quantification of liver inflammation was performed according to the periportal hepatitis using the following scales: 0, no inflammation; 1, mild inflammation (only a focal area of periportal hepatitis); 2, moderate inflammation (periportal hepatitis with several foci around the circumference); and 3, severe inflammation (periportal hepatitis with nearly surrounding the entire circumference; 1-3). Both the liver fibrosis and inflammation were evaluated by a “blinded” liver pathologist. For immunohistochemistry (IHC), tumor sections were stained with the appropriate antibodies, and both the intensity and extent of immunostaining were taken into consideration when analyzing the data. (4).

**Apoptosis analyses**

For apoptosis analysis, 1×10^6^ HCC cells were placed into a 6-well plate, followed by 5 μM sorafenib or 100 μM S3I-201 with or without TNF-α and IL-6. After incubation for 48 h, adherent cells were collected and washed twice with PBS. The cells were stained with Annexin V-FITC (AV) and propidium iodide (PI), and the percentage of Annexin V-FITC-positive cells was measured by flow cytometry. For terminal deoxynucleotidyl transferase–mediated deoxyuridine triphosphate nick-end labeling (TUNEL) assay, apoptosis in tumor sections was assessed using riboAPO™ One-Step TUNEL Apoptosis Kit according to the manufacturer instructions.

**Reference:**

1. Goodman ZD. Grading and staging systems for inflammation and fibrosis in chronic liver diseases. Journal of Hepatology 2007;47:598-607.

2. Deng YR, Ma HD, Tsuneyama K, *et al*. STAT3-mediated attenuation of CCl_4_-induced mouse liver fibrosis by the protein kinase inhibitor sorafenib. J Autoimmun 2013;46:25-34.

3. Mohs A, Kuttkat N, Reissing J, *et al*. Functional role of CCL5/RANTES for HCC progression during chronic liver disease. J Hepatol 2017;66:743-753.

4. He L, Zhou X, Qu C, *et al*. Musashi2 predicts poor prognosis and invasion in hepatocellular carcinoma by driving epithelial-mesenchymal transition. J Cell Mol Med 2014;18:49-58.

5. Mederacke I, Dapito DH, Affo S, *et al*. High-yield and high-purity isolation of hepatic stellate cells from normal and fibrotic mouse livers. Nat Protoc 2015;10:305-315.

**Supplementary Table 1. Real-time polymerase chain reaction primers**

| Gene | Forward primer (5′ to 3′) | Reverse primer (5′ to 3′) |
| --- | --- | --- |
| *E-cadherin* | TCGACACCCGATTCAAAGTGG | TTCCAGAAACGGAGGCCTGAT |
| *Vimentin* | TGGCCGACGCCATCAACACC | CACCTCGACGCGGGCTTTGT |
| *N-cadherin* | GCGCGTGAAGGTTTGCCAGTG | CCGGCGTTTCATCCATACCACAA |
| *GAPDH* | GCCCTTATGTCGGAGCTGAAGA | GTTGCGGTGCAGGTAGTCCA |
| mouse *TNF-α* | CCCTCACACTCAGATCATCTTCT | GCTACGACGTGGGCTACAG |
| mouse *IL-1β* | TGCCACCTTTTGACAGTGATG | TGATGTGCTGCTGCGAGATT |
| mouse *IL-6* | TAGTCCTTCCTACCCCAATTTCC | TTGGTCCTTAGCCACTCCTTC |
| mouse *IL-8* | CAAGGCTGGTCCATGCTCC | TGCTATCACTTCCTTTCTGTTGC |
| mouse *IL-12* | TGGTTTGCCATCGTTTTGCTG | ACAGGTGAGGTTCACTGTTTCT |
| mouse *IL-17* | TTTAACTCCCTTGGCGCAAAA | CTTTCCCTCCGCATTGACAC |
| mouse *IL-18* | GACTCTTGCGTCAACTTCAAGG | CAGGCTGTCTTTTGTCAACGA |
| mouse *Nos2* | GTTCTCAGCCCAACAATACAAGA | GTGGACGGGTCGATGTCAC |
| mouse *Cox2* | TTCAACACACTCTATCACTGGC | AGAAGCGTTTGCGGTACTCAT |
| mouse *Vcam1* | AGTTGGGGATTCGGTTGTTCT | CCCCTCATTCCTTACCACCC |
| mouse *Acta2* | TCCCTGGAGAAGAGCTACGAACT | AAGCGTTCGTTTCCAATGGT |
| mouse *Col1a1* | CCTGGCAAAGACGGACTCAAC | GCTGAAGTCATAACCGCCACTG |
| mouse *TIMP1* | CCAGAACCGCAGTGAAGAGT | GTACGCCAGGGAACCAAGAA |
| mouse *E-cadherin* | CAGGTCTCCTCATGGCTTTGC | CTTCCGAAAAGAAGGCTGTCC |
| mouse *Vimentin* | CGTCCACACGCACCTACAG | GGGGGATGAGGAATAGAGGCT |
| mouse *N-cadherin* | AGCGCAGTCTTACCGAAGG | TCGCTGCTTTCATACTGAACTTT |
| mouse *GAPDH* | GGACCTCATGGCCTACATGG | TAGGGCCTCTCTTGCTCAGT |
